# Correlates of early reproduction and apparent fitness consequences in male Soay sheep

**DOI:** 10.1002/ece3.10058

**Published:** 2023-05-07

**Authors:** Elisabeth G. Chapman, Jill G. Pilkington, Josephine M. Pemberton

**Affiliations:** ^1^ School of Biological Sciences, Institute of Ecology and Evolution University of Edinburgh Edinburgh UK

**Keywords:** early life reproduction, life history, Soay sheep, trade‐offs

## Abstract

Life history trade‐offs are ubiquitous across species and place constraints on the timing of life history events, including the optimal age at first reproduction. However, studies on lifetime breeding success of male mammals are rare due to sex‐biased dispersal and the requirement for genetic paternity inferences. We studied the correlates and apparent fitness consequences of early life reproduction among males in a free‐living population of Soay sheep (*Ovis aries*) on St Kilda, Scotland. We investigated the factors associated with early breeding success and the apparent consequences of early success for survival and future reproduction. We used genetic paternity inferences, population data, and individual morphology measurements collected over 30 years. We found that individuals born in years with low‐density population size had the highest early life breeding success and singletons were more likely to be successful than twins. Individuals that bred successfully at 7 months were more likely to survive their first winter. For individuals that survived their first winter, early breeding success was not associated with later breeding success. As individual heterogeneity affects breeding success, we believe that variation in individual quality masks the costs of early reproduction in this population. Our findings provide no evidence for selection for delayed age at reproduction in male Soay sheep.

## Graphical abstract

We investigated the factors associated with early breeding success in male Soay sheep and the consequences of this for survival and future reproduction. We found that individuals born into low‐density populations had the highest early life breeding success. Individuals that bred in their first year were more likely to survive their first winter but breeding early had no effect on their future reproductive success.

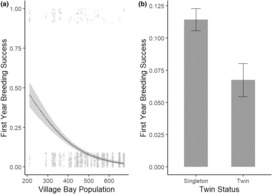

## 1 INTRODUCTION

Life history theory states that trade‐offs between investment in life history stages will occur when a favorable change in one trait is associated with a detrimental change in another (Reznick, 1985; Stearns, 1989). This places constraints on individuals' timing of investment in certain life stages (Brommer, 2007; Iwasa & Levin, 1995). Without such constraints, organisms would start reproducing immediately after birth and live indefinitely (Law, 1979). For example, early reproduction can decrease survival and future fecundity (Bell, 1980; Nussey et al., 2006). In species with indeterminate growth such as eastern gray kangaroos (*Macropus giganteus*; Gélin et al., 2016) and some fishes and reptiles (Heino & Kaitala, 1996), breeding early restricts growth. Balancing these trade‐offs between age and size at maturity forces individuals to delay reproduction until they are large enough. Reproduction can also affect survival as demonstrated in female rhesus macaques (*Macaca mulatta*; Blomquist, 2009) and red deer (*Cervus elaphus*) where rearing a calf decreases the probability a female will survive the subsequent winter (Clutton‐Brock et al., 1983; Froy et al., 2016).

Understanding when animals begin breeding is a fundamental question in ecology (Brommer et al., 1998; Cole, 1954). All animals face life history trade‐offs, and the optimal age at first breeding can be predicted by balancing the costs and benefits associated with reproduction (Reznick, 1985; Stevenson et al., 2004). There is high inter‐ and intraspecies variation in age at first breeding (Fay et al., 2016) and understanding the costs and benefits that lead to this diversity is vital in understanding life history evolution. In general, natural selection favors a “live fast, die young” approach in males, favoring the maximization of reproductive output (Bateman, 1948; Vinogradov, 1998). Sexual selection acts on males, causing them to develop characteristics for both pre‐ and postcopulatory competition which represent their own trade‐offs. Competition for mates has led to the evolution of male secondary sex characteristics such as weaponry and elaborate features (Andersson, 1986; Robinson et al., 2006). In promiscuous populations, postcopulatory competition in the form of sperm competition within the female is important, with sexual selection acting on testes size, and therefore sperm count. Both these phenomena represent another trade‐off for males, whereby investing energy and resources in developing such characteristics may reduce their investment in early life survival, reproduction, or ejaculate quality (Bonduriansky et al., 2008; Simmons et al., 2017; Travers et al., 2015). There is evidence across taxa demonstrating aging costs associated with an investment in both pre‐ and postcopulatory competition which is not avoided by investing in reproduction early in life (Lemaître et al., 2020). However, in male mammals, there are relatively few studies examining reproductive trade‐offs (for an exception see Lemaître et al. (2014)), perhaps because they are usually the more dispersive sex (Archer et al., 2022).

There is also variation in *which* individuals breed when they are able to. Differences in breeding success can be attributed to population‐, social‐, or individual‐level factors. Population‐level factors might include factors such as the ecological conditions under which an individual is growing or the operational sex ratio. Social factors can be seen in cooperative breeders. For example, social vertebrates often have hierarchies constructed around a dominant breeding pair (Sparkman et al., 2011) and extremes are reached in eusocial insects with sterile individuals (Anderson, 1984). Individual heterogeneity can also affect the breeding success of individuals. For example, in strongly polygynous species, where male–male competition for mates is intense, early breeding success may be associated with individual variation in growth and development (Ritchot et al., 2021; Wilson & Nussey, 2010).

Here, we examine the cost and benefits of early reproduction in a wild ungulate population—the Soay sheep (*Ovis aries*) of St Kilda. Both sexes of Soay sheep mature early, with males participating in their first rut at 7 months old (Stevenson & Bancroft, 1995) and some females becoming pregnant at the same age. Soay sheep have a highly promiscuous seasonal rut in which juvenile males are sometimes able to obtain matings and sire offspring (Pemberton et al., 1999). Females are in estrous for ~24 h and may mate many times with multiple males (Clutton‐Brock, Pemberton, et al., 2004). Large, dominant males sequester individual females in “consorts” lasting several hours and copulate many times (Clutton‐Brock, Pemberton, et al., 2004). Males seen holding a consort are more likely to sire offspring than other males (Coltman, Bancroft, et al., 1999), however, as a result of frequent mating, large, dominant males become sperm depleted (Preston et al., 2001). This allows subordinate males, including those with small horns and juveniles, which obtain matings via chasing females rather than holding consorts, to outcompete dominant males through sperm competition, especially towards the end of the rut (Preston et al., 2003) and so sire offspring (Pemberton et al., 1999). These chases involve estrous females being pursued by multiple males attempting to mate with them and are energetically costly for both sexes (Coltman, Bancroft, et al., 1999; Stevenson et al., 2004). The cost of rutting activity to juvenile males was investigated experimentally by temporary chemical castration, following which treated males survived the ensuing winter better than controls (Stevenson & Bancroft, 1995). Relative testes size is known to be related to promiscuity and sperm competition in numerous taxa (reviewed by Parker et al., 1997), including Soay sheep (Preston et al., 2003), which have the largest testes to body size ratio of any sheep (Lincoln, 1989). Soay sheep face high overwinter mortality and this selects for both precocial maturity and increased early reproductive effort (Heino & Kaitala, 1996; Stevenson & Bancroft, 1995) and raises the question of whether early reproduction carries costs to survival and future reproduction.

Our previous analyses of juvenile breeding success in the study population were based on paternity inference via a panel of microsatellites and the parentage inference software CERVUS which was underpowered for determining paternity in that only a proportion of paternities could be resolved, often at confidence as low as 80%, particularly in years with many competing males (Pemberton et al., 1999). Here, we use over 30 years of data and a much more powerful paternity inference system to understand the correlates and apparent consequences of early life reproduction in male Soay sheep. We ask the following questions:

1. What factors are associated with breeding success in juvenile males and do individual factors such as body size interact with population factors to affect breeding success?
2. What consequences does early life reproduction have for survival and future reproduction?

## 2 METHODS

### 2.1 Study population and data collection

The Soay sheep of St Kilda have lived unmanaged on the island of Hirta since they were introduced there in 1934 after the last human inhabitants of St Kilda left (Clutton‐Brock & Pemberton, 2004). The population fluctuates in density between years due to over‐compensatory density dependence combined with winter weather conditions (Clutton‐Brock, Grenfell, et al., 2004; Coulson, 2001; Grenfell et al., 1992). The majority of mortality occurs over winter and is biased toward juveniles and males, who go into winter in poor condition after the intense rutting season (Stevenson & Bancroft, 1995; Stevenson et al., 2004). After winters of high mortality, the population is heavily female‐biased (Clutton‐Brock, Grenfell, et al., 2004). This variability means that in high‐density years, there is more intense male–male competition for mates in the rut, with a large population and relatively even sex ratio, while in low‐density years, there is a higher ratio of females to males and competition is lower (Preston et al., 2003; Stevenson & Bancroft, 1995).

The field data and samples were collected by research teams on St Kilda between 1986 and the spring of 2020. Data are collected three times each year; in late winter/early spring, summer, and autumn (Clutton‐Brock & Pemberton, 2004). Ten study area censuses are conducted on each expedition, yielding population size and sex ratio data. Population size and female:male sex ratio are inversely correlated with *R* = −.81 (Table S2). In spring, mortality checks are carried out to determine which individuals died over winter. Here, we used data only from males that died in the Village Bay study area during the study period. Although this means that we exclude data for the relatively few males that disperse from the study area, we do explicitly test for differences in early reproductive success between males born within or outside the study area. Lambs are caught soon after birth, weighed, sampled for genetic analysis, and tagged for lifelong identification. In August as many individuals as possible are caught, tagging any untagged individuals, and collecting up‐to‐date measurements of body size including horn length, horn basal circumference, hindleg length, testes length, testes circumference, and body weight. Individuals born as twins weigh less than singletons at birth, and this is still the case in August and all August body size measures are positively correlated (see Table S1 for correlation matrix). Soay sheep rut in November; at this time, 4–5 censuses of the study area are carried out per day to note the identity of pairs in consorts. During this time, any immigrant males from elsewhere on the island are also captured by darting, measured, sampled, and tagged.

### 2.2 Genetic paternity analysis

Behavioral observations in the rut are a poor guide to the paternity of specific lambs due to the promiscuity of both sexes and intense sperm competition (Coltman, Bancroft, et al., 1999; Pemberton et al., 1999) so paternities were determined genetically. Paternity was inferred using 431 single nucleotide polymorphisms (SNPs) genotyped in virtually all study area individuals and the R pedigree reconstruction package SEQUOIA (Bérénos et al., 2014; Huisman, 2017). This approach provides highly accurate paternity assignments, with an error rate of <0.1% (Huisman, 2017). A small number of paternities involving individuals not SNP genotyped were inferred using microsatellite markers and the R package MasterBayes (Hadfield et al., 2006; Morrissey et al., 2012).

### 2.3 Data analysis

All data analysis was conducted in R 4.0.3 (R Core Team, 2020). We created models with the lme4 package (Bates et al., 2015). Response and predictor variables are shown in Table 1. When fitted, a random term for the birth year did not affect the results so we exclude it here. We retained maximal models for our main interpretation but also used a process of model simplification by sequentially removing the least significant terms from the model to determine the most important factors.

**TABLE 1:** Response and predictor variables used in the analyses.

	Description
Response variable	
Success	Bred successfully in first year (1) or not (0)
First‐year survival	Survived first winter (1) or not (0)
Subsequent offspring	Count of offspring after first year (in males that survived the first year)
Predictor variable	
Population size	Total number of sheep in study area in birth year
Population sex ratio	Sex ratio female: male in study area in birth year
Mother known	Mother known (1—resident) or not (0—immigrant)
Twin status	Singleton (0), twin (1)
Horn type	Horn type scurred (1), or normal (3)
Horn circumference	Circumference at the base of horn (mm)
Hindleg	Hindleg length (metatarsal) (mm)
Testes circumference	Testes circumference (mm)
Weight	Weight (kg)
Success	Bred successfully in first year (1) or not (0)

*Note*: Measurements of individual body size were taken in August of the male's first year.

#### 2.3.1 What factors are associated with reproductive success in male lambs?

Juvenile males rarely obtained more than one paternity, so breeding success was analyzed as a binary response with individuals assigned 1 (successful) or 0 (unsuccessful) (Table 1). We created three generalized linear models (GLMs) using a binomial distribution, analyzing which population‐ and individual‐level factors were associated with mating success in male lambs (Table 2). The three models included increasing amounts of information about individual males but consequently also decreasing sample sizes. Model 1 focused on annual variation in population size and sex ratio and whether there was a difference in success between resident and immigrant juvenile males; it included all individuals in the study area at the time of the rut. The breeding success of male lambs is likely to be associated with variation in the conditions they experience in their first year as well as individual variation in growth and development. Coltman, Smith, et al. (1999) and Pemberton et al. (1999) found that males born into a low‐density population are more likely to breed successfully in their first year. This is presumably due to sex‐biased mortality (Coltman, Smith, et al., 1999), creating a high ratio of females following a harsh winter and reducing the intensity of male competition (Stevenson & Bancroft, 1995).

**TABLE 2:** Models used in analysis.

Model	Response variable	Maximal model terms
1	Success (1/0)	Population size, Population sex ratio, Mother known
2	Success (1/0)	Population size, Population sex ratio, Twin status, Horn type
3	Success (1/0)	Population size, Twin status, Horn circumference, Hindleg, Testes circumference, Weight, Weight × Population size, Testes circumference × Population size
4	Survival (1/0)	Success, Population size, Twin status, Testes circumference, Weight, Horn type, Weight × Population size, Testes circumference × Population size
5	Subsequent offspring	Success, Population size, Twin status, Testes circumference, Weight, Horn type, Weight × Population size, Testes circumference × Population size

Model 2 only included individuals born in the study area and used individual‐level data available from the lambing season, namely twin status, and horn type. Individuals born as twins weigh less than singletons, show slower growth rates, and must share maternal resources between two individuals (Clutton‐Brock et al., 1992; Clutton‐Brock, Pemberton, et al., 2004). We therefore might expect twins to have lower first‐year breeding success than single‐born males. Male Soay sheep have two genetically determined horn types: either “normal” (coded as 3) or smaller, deformed horns known as “scurred” (coded as 1). Females have a third horn type (“polled”: coded as 2) which males do not have. Males with scurred horns show increased survival but cannot compete in physical conflicts with normal horned males and have reduced lifetime reproductive success (Johnston et al., 2013).

Individual factors, such as horn type and weight, are known to affect breeding success of males throughout their lives (Preston et al., 2003). Heavier males and those with normal horns obtain higher lifetime breeding success (Johnston et al., 2013; Robinson et al., 2006; Stevenson et al., 2004). To test these, model 3 only included individuals that were caught and phenotyped in their first August. This model included two interaction terms, one between weight and population and another between testes circumference and population. These interaction terms investigated whether the relationships found between body size measures and success varied with density. The terms included in models 1–3 are detailed in Table 2.

#### 2.3.2 What effect does early life reproduction have on survival and future reproduction?

In model 4, we used a binomial GLM to determine factors affecting first‐year survival. We created a binary response variable by assigning individuals 1 (survived) or 0 (died) for survival over their first winter. We asked what population and individual characteristics, including early success, were associated with survival as shown in Table 2. We again investigated interactions between both body size and testes size with population density to investigate whether relationships between body size and survival varied with density.

To quantify the effects of reproducing early on future reproduction, we computed the number of offspring sired *after* the first year, for those males that survived their first winter. We used a negative binomial GLM using the MASS package (Venables & Ripley, 2002) to determine the factors associated with reproductive success following individuals' first rut. We asked whether first‐year success predicted subsequent reproductive success alongside population and individual characteristics measured in the first year of life. Again, interaction terms were included between weight and population size, and testes circumference and population size (Table 2).

## 3 RESULTS

Overall, over the years 1986–2019, 221 of 2047 juvenile males (10.8%) obtained at least one paternity in their first rut.

### 3.1 What factors are associated with reproductive success in male lambs?

**FIGURE 1:**
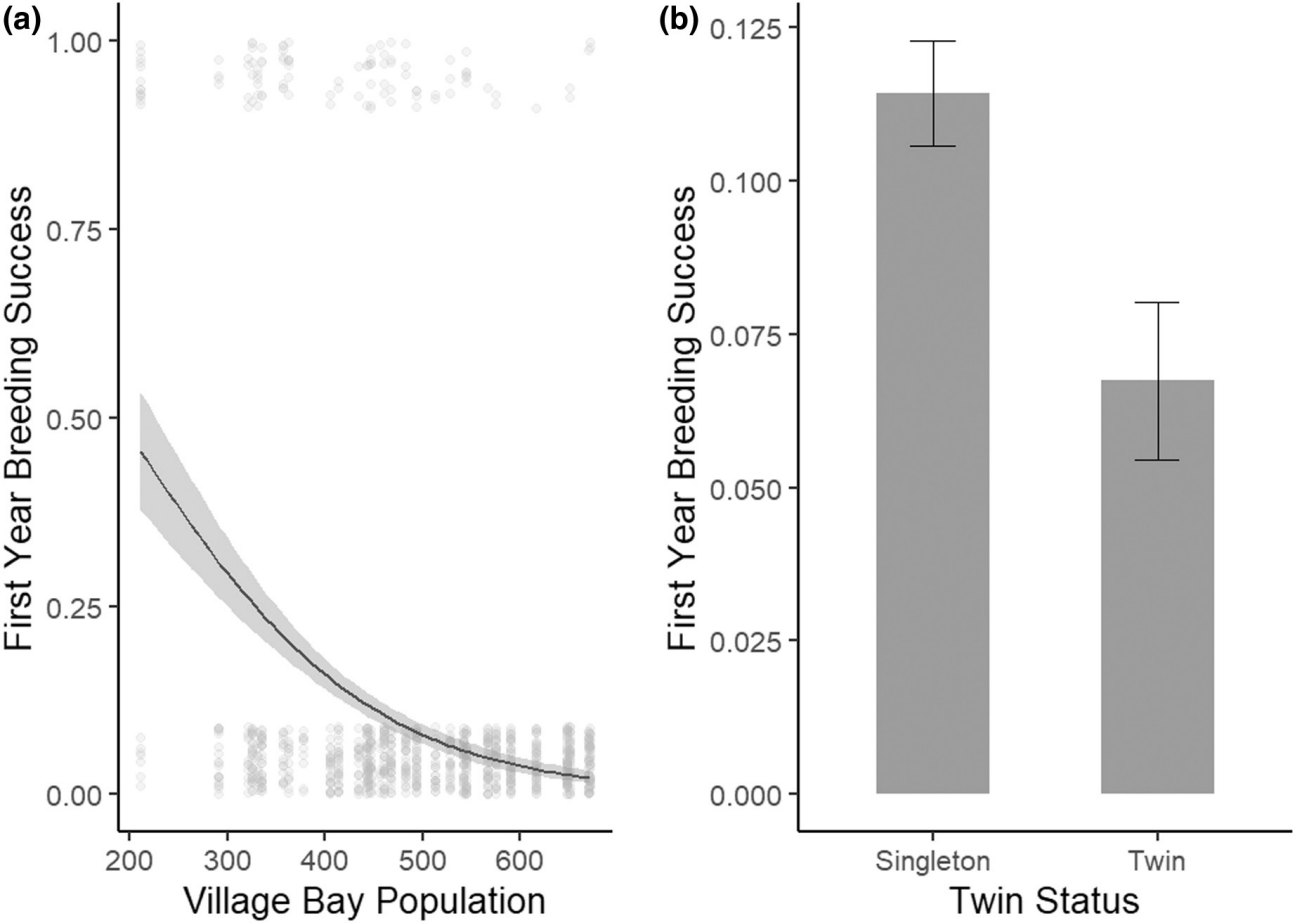
Factors associated with first‐year breeding success. (a) Association between population size and breeding success from model 1 with dots representing individuals that either bred successfully in their first year (1) or did not (0) and shading showing 95% confidence intervals. (b) The association between twin status and breeding success from model 2 with error bars showing standard error.

In model 1, we found that males born in low‐density years had higher first‐year breeding success than those born into high‐density years (Table 3, Figure 1a). Population sex ratio and immigrant status were not significant in the maximal model nor were they significant in any model simplification (Table S3).

**TABLE 3:** Model output showing coefficients from model 1: Juvenile breeding success for all individuals in the Village Bay study area at the time of the rut.

	Estimate	SE	*Z* value	*p* Value
(Intercept)	0.347	0.997	0.348	.728
Mother known	0.056	0.208	0.268	.789
Population	−0.007	0.001	−6.316	<.001
Sex ratio	0.297	0.253	1.172	.241

*Note*: *N* = 2047. Null deviance: 1401.1 on 2046 degrees of freedom. Residual deviance: 1248.0 on 2043 degrees of freedom.

In model 2, we found that in addition to the population size effect, males born as twins had lower breeding success in their first year than those born as singletons (Table 4, Figure 1b). Here, horn type and sex ratio were not significant in the maximal model, nor in any model simplification (Table S3).

**TABLE 4:** Model output showing coefficients from model 2: Juvenile breeding success for all individuals born into the Village Bay study area.

	Estimate	SE	*Z* value	*p* Value
(Intercept)	0.458	1.142	0.401	.688
Twin status	−0.559	0.228	−2.450	.014
Horn type	0.002	0.133	0.014	.989
Population	−0.007	0.001	−5.761	<.001
Sex ratio	0.299	0.279	1.074	.283

*Note*: *N* = 1776. Null deviance: 1186.9 on 1775 degrees of freedom. Residual deviance: 1055.2 on 1771 degrees of freedom.

In model 3, we found that early reproduction is not predicted by any measured term in the maximal model (Table 5). However, under model simplification population size and testes circumference were the most important predictors of first‐year breeding success (Table S3). Weight also predicted breeding success, but only if testes circumference was not included (Table S4). Neither twin status nor horn circumference predicted breeding success in the maximal model or any simplified version.

**TABLE 5:** Model output showing coefficients from model 3: Juvenile breeding success for all individuals caught in their first August.

	Estimate	SE	*Z* value	*p* Value
(Intercept)	−1.881	5.731	−0.328	.743
Weight	−0.587	1.507	−0.106	.916
Horn circ.	−0.063	0.338	−0.187	.852
Testes circ.	0.187	0.439	0.116	.908
Hindleg	0.288	0.365	0.789	.430
Population	−0.202	0.137	−1.476	.140
Twin status	−0.323	1.588	−0.055	.956
Weight × Population	0.040	0.163	0.247	.805
Testes circ. × Population	0.012	0.048	0.261	.794

*Note*: *N* = 805. Null deviance: 480.34 on 804 degrees of freedom. Residual deviance: 415.61 on 796 degrees of freedom.

### 3.2 Is early life reproduction associated with survival and future reproduction?

#### 3.2.1 Association with survival

**FIGURE 2:**
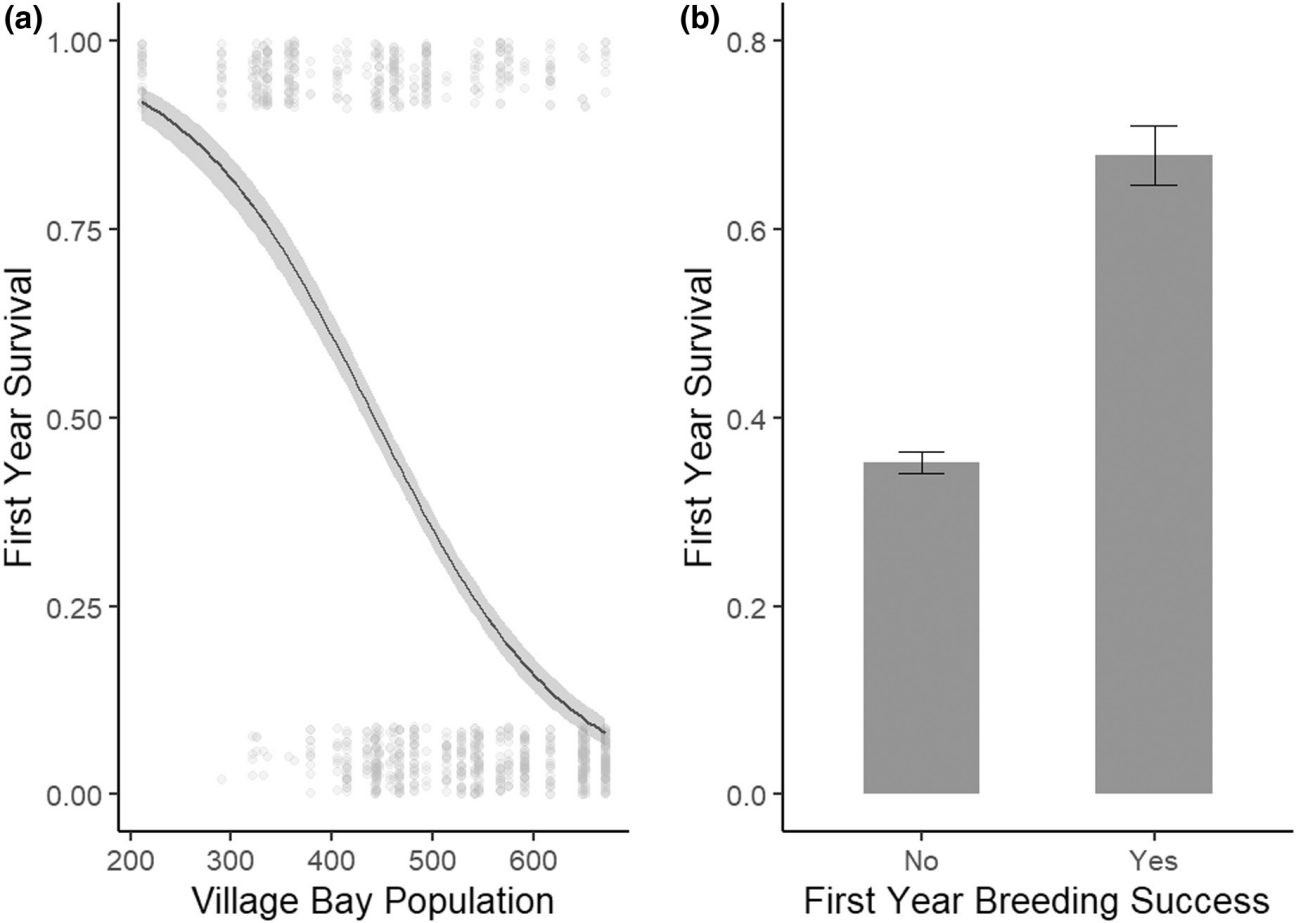
Factors associated with first‐year survival of male lambs (model 4). (a) The relationship between population size and first‐year survival. Dots represent individuals who either survived (1) or died (0) in their first winter, and shading represents 95% confidence intervals. (b) The relationship between breeding success and first year survival with error bars showing standard error.

In model 4, as expected, we found that individuals were more likely to survive through low‐density winters (Table 6, Figure 2a). We also found that individuals that bred successfully in their first rut were more likely to survive their first winter (Table 6, Figure 2b).

**TABLE 6:** Model output showing coefficients from model 4: Factors associated with first‐year survival.

	Estimate	SE	*Z* value	*p* Value
(Intercept)	2.767	2.993	3.401	<.001
Success	2.061	1.023	2.014	.044
Weight	0.289	1.087	0.072	.942
Testes circ.	−0.409	0.311	−1.314	.189
Population	−0.404	0.313	−4.738	<.001
Twin status	1.052	0.910	1.156	.248
Horn type	0.899	0.532	0.460	.646
Weight × Population	0.108	0.112	0.963	.336
Testes circ. × Population	0.036	0.032	1.110	.267

*Note*: *N* = 856. Null deviance: 1092.01 on 855 degrees of freedom. Residual deviance: 796.16 on 847 degrees of freedom.

#### 3.2.2 Association with future reproduction

Following an individual's first year, in model 5, we found no relationship between first‐year breeding success, nor any individual‐level factors, and the number of subsequent offspring (Table 7). However, we found that population size in the year of birth was negatively associated with the number of subsequent offspring an individual had. First‐year breeding success did not explain significant variation in the number of subsequent offspring, nor was it retained following model simplification (Table S3).

**TABLE 7:** Model output showing coefficients from model 5: Factors associated with lifetime reproductive success.

	Estimate	SE	*Z* value	*p* Value
(Intercept)	6.864	3.553	1.932	.053
Success	0.542	1.234	0.119	.905
Testes circ.	−0.170	0.343	−0.497	.619
Weight	−0.728	1.203	−0.605	.545
Population	−0.249	0.406	−2.262	.024
Horn type	1.163	0.794	1.464	.143
Twin status	−0.189	1.315	−0.039	.969
Weight × Population	0.072	0.140	0.513	.608
Testes circ. × Population	0.037	0.039	0.938	.348

*Note*: *N* = 287. Null deviance: 280.69 on 286 degrees of freedom. Residual deviance: 269.68 on 278 degrees of freedom.

## 4 DISCUSSION

Our analyses show that both population and individual factors are associated with the probability that a juvenile male Soay sheep sires a lamb in his first rut. They also show that success in the first rut is associated with *increased* probability of survival over the first winter and is not traded off against breeding success over the rest of the lifetime.

### 4.1 What factors are associated with reproductive success in juvenile males?

In models 1 and 2, we found that male lambs born into high‐density years had lower breeding success. This is consistent with the effect of first‐year population size on lifetime reproductive success demonstrated by Coltman, Smith, et al. (1999). As overwinter mortality is male‐biased, population crashes result in a female‐biased population the following year (Stevenson & Bancroft, 1995). This causes a rut with low competition and therefore, higher mating success of male lambs because there are more females not in consort that juvenile rams can chase and mate with. In high‐density years, there is intense competition for females during the rut with few juvenile males obtaining matings. From this line of argument, one would predict that the sex ratio would be associated with juvenile male success, but this was not the case when population size was included in the model, suggesting additional effects due to population size are at work. One possibility is that as well as capturing sex ratio, population size captures year‐level effects of male condition: in low‐density years, juvenile males may be better resourced to compete. Density‐dependent costs associated with reproduction have also been demonstrated in a long‐term study of bighorn sheep (*Ovis canadensis*; Festa‐Bianchet et al., 1998). These authors comment on conflicting trends in different mammal populations and suggest that reproductive costs are associated with poor condition individuals and high‐density populations, consistent with our results.

Model 1 demonstrated that immigrant males were no more successful than males born in the study are. We tested immigrant status because there is evidence from other ruminants that males that disperse early are better grown (Clutton‐Brock et al., 2002), but this did not result in greater juvenile breeding success in this case. This result also suggests to us that our inability to track the small number of males that permanently emigrate from the study should not seriously bias our findings.

Model 2 showed that individuals born as twins were less successful than those born as singletons. Soay twins are born lighter than single lambs (Clutton‐Brock et al., 1992) and are still lighter when weighed in August, as the mother has to distribute her resources between two offspring leading to slower growth rates (Clutton‐Brock, Pemberton, et al., 2004). The lower success of male twins is the first indication that aspects of body size are important predictors of early breeding success.

In model 3, we found that individuals with greater testes circumference or higher weight were more likely to sire a lamb, although these two variables displaced each other in the model, and each was only significant under model simplification. Meanwhile twin status dropped out of the simplified model. This confirms the idea from model 2 that size matters, with the reduced body size due to being a twin being replaced by actual size in model 3. Testes circumference and body size are strongly correlated in juvenile males (Table S1) so it is not surprising that both cannot be fitted in the simplified model at the same time, but testes size was the better predictor of success. This is consistent with the prevalence of sperm competition in the population (Preston et al., 2003). The importance of testes size in sperm competition has been demonstrated in many taxa (Parker et al., 1997) including primates (Møller, 1988), frogs (Jennions & Passmore, 2008), and other ungulates (Ginsberg & Rubenstein, 1990). Large testes are associated with high testosterone levels which increases muscle mass, reproductive behavior and weaponry in Soay sheep (Preston et al., 2012). Therefore, individuals with large testes are more likely to copulate and pass on many high‐quality sperm to receptive females and this is especially likely toward the end of the rut as older males become sperm depleted (Preston et al., 2001). However, the fact that these were only significant following model simplification demonstrates that many aspects of individual heterogeneity likely come together to affect early life reproductive success and identifying individual effects while accounting for covariation remains a challenge.

We found no relationship between horn size and first‐year breeding success in model 3. Scurred adults with small horns have decreased consort and paternity success compared to individuals with normal horns (Preston et al., 2001, 2003; Stevenson et al., 2004). Juvenile males, even if they have normal horns, have much smaller horns than most of the older males they are competing with and in this respect are like scurred males. Our results suggest that for these juveniles, individual condition and capacity for sperm competition are more important than weaponry in obtaining paternities. Horns allow males to defend females in consorts during the rut, but juvenile males were only involved in 333 of 11,273 (2.9%) observed consorts over the study period. It seems likely that horn type or size is of little importance in juvenile males as they do not rely on consort success for breeding, instead chasing estrous females to copulate. Across taxa, there are examples of subordinate males opting for alternative mating tactics when they cannot compete with large, dominant males. For instance, black gobies (*Gobius niger*) demonstrate size‐dependent mating tactics with smaller males mimicking females to obtain sneak matings by releasing sperm at the same time as larger, parental males (Mazzoldi & Rasotto, 2002). Age‐dependent mating tactics have been demonstrated in other polygynous ungulates, such as Alpine ibex (*Capra ibex*) where young males are most likely to sire offspring through sneak tactics (Willisch et al., 2012).

### 4.2 Is early life reproduction associated with survival and future reproduction?

In contrast to predictions made by life history theory, in model 4, we found a positive effect of reproducing early on first‐year survival. As expected, survival was also higher for individuals born into low‐density years and higher for individuals that were heavier in their first year. In model 5, we asked whether early breeding success and other factors prevalent in the first year of life predicted subsequent breeding success among those males that survived the first year. Only population size in the year of birth predicted subsequent reproduction, with individuals born into low‐density years having the highest lifetime reproduction. We found no relationship between breeding early and the number of offspring following an individual's first year.

The lack of detectable costs to breeding successfully in the first year, in terms of either first winter survival or subsequent breeding success is not entirely unexpected. Though life history trade‐offs have been demonstrated experimentally (Cox et al., 2010; Hanssen et al., 2005), in many studies on natural populations (van Noordwijk & de Jong, 1986), including our own, positive correlations between life history events are found instead of trade‐offs. The most likely explanation is that variation in individual quality is masking any costs of early reproduction, so high‐quality individuals can be successful at mating and at survival. The fact that heavier males or males with large testes were more successful as juveniles (under model simplification) and successful individuals were more likely to survive their first winter is an indication that this might be the case.

In conclusion, even though the experimental evidence is that juvenile males would survive bad winters better if they did not try to get matings (Stevenson & Bancroft, 1995) our correlational study provides little evidence for a cost of early reproduction in male Soay sheep, due to masking individual variation in quality. In the face of unpredictable (to the sheep) variation in first winter mortality, selection favors early reproduction across the board.

## AUTHOR CONTRIBUTIONS

**Elisabeth G. Chapman:** Conceptualization (supporting); formal analysis (lead); writing – original draft (lead); writing – review and editing (equal). **Jill G. Pilkington:** Data curation (lead). **Josephine M. Pemberton:** Conceptualization (lead); formal analysis (supporting); funding acquisition (lead); project administration (lead); supervision (lead); writing – original draft (supporting); writing – review and editing (equal).

## CONFLICT OF INTEREST STATEMENT

The authors declare no competing interests.

## Supporting information


Tables S1–S4.

## Data Availability

The data that support the findings of this study are openly available in Dryad at https://doi.org/10.5061/dryad.wm37pvmsh.
