# Supplementary material for: Correlates of early reproduction and apparent fitness consequences in male Soay sheep
Source: Ecol Evol. 2023 May 7;13(5):e10058. doi: 10.1002/ece3.10058 (PMC10164647; doi:10.1002/ece3.10058)
Supplement: Supplementary file 1 — Tables S1–S4. [file ECE3-13-e10058-s001.docx]

**Supplementary information**

**Table S1.** Correlation matrix showing relationship between individual body size measurements of males caught in their first August.

|  | **Testes Length** | **Testes Circ.** | **Horn Circ.** | **Horn Length** | **Hindleg** | **Foreleg** | **Weight** | **Horn Type** |
| --- | --- | --- | --- | --- | --- | --- | --- | --- |
| **Twin Status** | -0.2238 | -0.2985 | -0.1526 | -0.1725 | -0.3664 | -0.2487 | -0.3468 | -0.0130 |
| **Horn Type** | -0.0042 | -0.0116 | 0.4810 | 0.5727 | -0.0264 | -0.0290 | 0.0262 |  |
| **Weight** | 0.7490 | 0.8037 | 0.3777 | 0.4429 | 0.8485 | 0.7043 |  |  |
| **Foreleg** | 0.5760 | 0.5832 | 0.2471 | 0.2932 | 0.8328 |  |  |  |
| **Hindleg** | 0.6778 | 0.7018 | 0.2392 | 0.3117 |  |  |  |  |
| **Horn Length** | 0.3621 | 0.3717 | 0.8896 |  |  |  |  |  |
| **Horn Circ.** | 0.3290 | 0.3036 |  |  |  |  |  |  |
| **Testes Circ.** | 0.8193 |  |  |  |  |  |  |  |

**Table S2.** Correlation matrix showing relationship between year-level demographic factors.

|  | **Total Population** | **Total Females** | **Total Males** | **Male Lambs** | **Female Lambs** |
| --- | --- | --- | --- | --- | --- |
| **Total F:M Ratio** | -0.8126 | -0.7184 | -0.8786 | -0.8265 | -0.6900 |
| **Female Lambs** | 0.8556 | 0.8711 | 0.8032 | 0.7275 |  |
| **Male Lambs** | 0.8436 | 0.8436 | 0.9164 |  |  |
| **Total Males** | 0.9827 | 0.9312 |  |  |  |
| **Total Females** | 0.9820 |  |  |  |  |

**Table S3.** Minimal models obtained following model simplification.

| Model | Response Variable | N | Retained Terms | Estimate | Std. Error | Z value | P vale |
| --- | --- | --- | --- | --- | --- | --- | --- |
| 1 | Success | 2047 | Intercept | 1.474 | 0.298 | 4.953 | <0.001 |
|  |  |  | Population | -0.008 | 0.001 | -11.472 | <0.001 |
| 2 | Success | 1778 | Intercept | 1.539 | 0.332 | 4.636 | <0.001 |
|  |  |  | Twin status | -0.603 | 0.227 | -2.661 | 0.008 |
|  |  |  | Population | -0.008 | <0.001 | -10.368 | <0.001 |
| 3 | Success | 858 | Intercept | -2.128 | 1.040 | -2.045 | 0.041 |
|  |  |  | Testes Circ. | 0.020 | 0.004 | 4.484 | <0.001 |
|  |  |  | Population | -0.008 | 0.001 | -6.824 | <0.001 |
| 4 | Survival | 856 | Intercept | 9.767 | 2.888 | 3.381 | <0.001 |
|  |  |  | Success | 2.006 | 1.028 | 1.952 | 0.051 |
|  |  |  | Weight | 1.063 | 0.770 | 5.078 | <0.001 |
|  |  |  | Testes Circ. | -0.623 | 0.202 | -3.084 | 0.002 |
|  |  |  | Population | -0.379 | 0.303 | -4.599 | <0.001 |
|  |  |  | Testes Circ.*Population | 0.058 | 0.020 | 2.843 | 0.004 |
| 5 | Subsequent Offspring | 287 | Intercept | 7.462 | 3.381 | 2.207 | 0.027 |
|  |  |  | Testes Circ. | -0.345 | 0.225 | -1.533 | 0.125 |
|  |  |  | Population | -0.240 | 0.391 | -2.256 | 0.024 |
|  |  |  | Testes Circ.*Population | 0.054 | 0.026 | 2.093 | 0.036 |

**Table S4.** Model output showing coefficients from minimal version of model 3 for juvenile breeding success if weight was retained rather than testes circumference.

|  | Estimate | Std. Error | Z value | P value |
| --- | --- | --- | --- | --- |
| (Intercept) | -1.808 | 0.844 | -2.142 | 0.032 |
| Weight | 0.197 | 0.043 | 4.570 | <0.001 |
| Population | -0.007 | 0.001 | -6.464 | <0.001 |

*N=1025*

*Null deviance: 650.91 on 1024 degrees of freedom*

*Residual deviance: 567.25 on 1022 degrees of freedom*
